# scPD: a Python package for inferring continuous population dynamics from single-cell snapshot data

**DOI:** 10.1093/bioadv/vbag188

**Published:** 2026-07-01

**Authors:** Yusong Yin, Hong Qi, Huan Hu

**Affiliations:** Complex Systems Research Center, Shanxi University, Taiyuan 030006,China; Complex Systems Research Center, Shanxi University, Taiyuan 030006,China; Shanxi Key Laboratory for Mathematical Techniques in Complex Systems, Shanxi University, Taiyuan 030006, China; Key Laboratory of Complex Systems and Data Science of Ministry of Education, Shanxi University, Taiyuan 030006, China; Institute of Applied Genomics, Fuzhou University, Fuzhou 350108, China; College of Biological Science and Engineering, Fuzhou University, Fuzhou 350108, China

## Abstract

**Summary:**

Quantitative inference of developmental dynamics from single-cell snapshot data is essential for disentangling differentiation and proliferation processes. The pseudodynamics framework provides a principled approach to this problem but lacks a scalable and user-friendly implementation for modern single-cell workflows. Here, we present scPD, a high-performance Python toolkit that implements and extends the pseudodynamics framework within the Scanpy ecosystem. scPD implements an efficient and scalable inference strategy, enabling the analysis of large-scale single-cell datasets with substantially reduced computational cost. This scalability enables kinetic parameter inference to be readily integrated into standard Python-based pipelines, facilitating quantitative characterization of population dynamics from time-resolved single-cell data.

**Availability and implementation:**

scPD is implemented in Python and is freely available as an open-source package on GitHub at https://github.com/yys-arch/scPD. Documentation and example notebooks are provided. The data used in this study are publicly available under DOI: 10.5281/zenodo.18337517.

## 1 Introduction

Inferring developmental dynamics from static single-cell transcriptomic snapshots is a central challenge in developmental and stem cell biology. Trajectory inference methods reconstruct continuous cell-state orderings and branching structures, providing a geometric view of developmental progression but lacking explicit temporal dynamics (Haghverdi *et al.* 2016, Saelens *et al.* 2019, Ranek *et al.* 2022). RNA velocity methods partially address this limitation by estimating local, instantaneous directions of cell-state transitions from splicing kinetics (La Manno *et al.* 2018, Bergen *et al.* 2020, Gayoso *et al.* 2024, Wang *et al.* 2025). However, velocity methods remain kinematic, characterizing instantaneous cell states rather than modeling how cell populations evolve over time in terms of probability distributions, stochasticity, and changes in cell abundance.

To move beyond geometric and kinematic descriptions, Fischer *et al.* introduced a pseudodynamics framework, which models cell differentiation as a stochastic process at the population level within a continuous state space (Fischer *et al.* 2019). By modeling the temporal evolution of cell-state probability densities with a Fokker-Planck equation, pseudodynamics explicitly captures directed differentiation, diffusive variability, and net population growth or loss, enabling quantitative inference of developmental dynamics from discrete snapshot data.

Building on this theoretical foundation, we present scPD, a scalable and general framework for inferring population dynamics from single-cell snapshots. By modeling drift, diffusion, and growth within a data-driven state space, scPD leverages the developmental progression captured in experimental snapshots to reconstruct the continuous temporal evolution of cell populations. Consequently, scPD extends pseudodynamics to modern single-cell settings, providing a solution for reconstructing developmental landscapes and population-level dynamics from static transcriptomic profiles.

## 2 Implementation

scPD provides a streamlined, end-to-end pipeline for inferring population dynamics from single-cell RNA sequencing (scRNA-seq) data. The workflow comprises five key steps. (i) State-space construction: Cells are ordered along a continuous state coordinate using diffusion pseudotime or user-defined trajectories. (ii) Population dynamics modeling: Population evolution along this state axis is described by a one-dimensional Fokker-Planck equation, where drift, diffusion, and net growth rates are modeled as smooth functions of the cell state. (iii) Parameter inference: Model parameters are estimated by minimizing the area distance between model-predicted cumulative distribution functions (CDFs) and empirical CDFs across experimental time points. (iv) Landscape reconstruction: From the inferred kinetic parameters, scPD derives a developmental potential landscape and reconstructs vector fields within the embedded state space. (v) Evaluation and visualization: The toolkit offers distribution-based metrics and standardized visualization tools to evaluate model fidelity and facilitate interpretation of the inferred dynamics.

To ensure scalability, scPD integrates a landmark-based acceleration scheme and supports both distribution-only and population-aware fitting modes. Detailed mathematical formulations and optimization procedures are provided in Supplementary Note 1.

## 3 Results

To demonstrate the capabilities of scPD, we perform both simulation-based benchmarking with known ground-truth dynamics and application to a time-resolved scRNA-seq dataset of mouse iPSC reprogramming (Schiebinger *et al.* 2019). The synthetic benchmark provides a controlled test of dynamical recovery, whereas the iPSC dataset tests whether the inferred dynamics recapitulate known features of reprogramming.

We first evaluate scPD using synthetic datasets generated from predefined Fokker-Planck dynamics with known diffusion coefficient D(s), drift velocity v(s), and net growth rate g(s). We considered two scenarios that mimic common developmental regimes: progressive differentiation toward a stable terminal state and a transient plasticity window during fate specification (Supplementary Note 2). In both scenarios, the fitted probability density functions matched the ground-truth distributions across time, with low area distance values at individual time points (Fig. S1a–d). The inferred kinetic profiles showed high Pearson correlations with the corresponding ground-truth functions, supporting accurate recovery of profile shapes under controlled settings (Fig. S1e).

**Figure 1:**
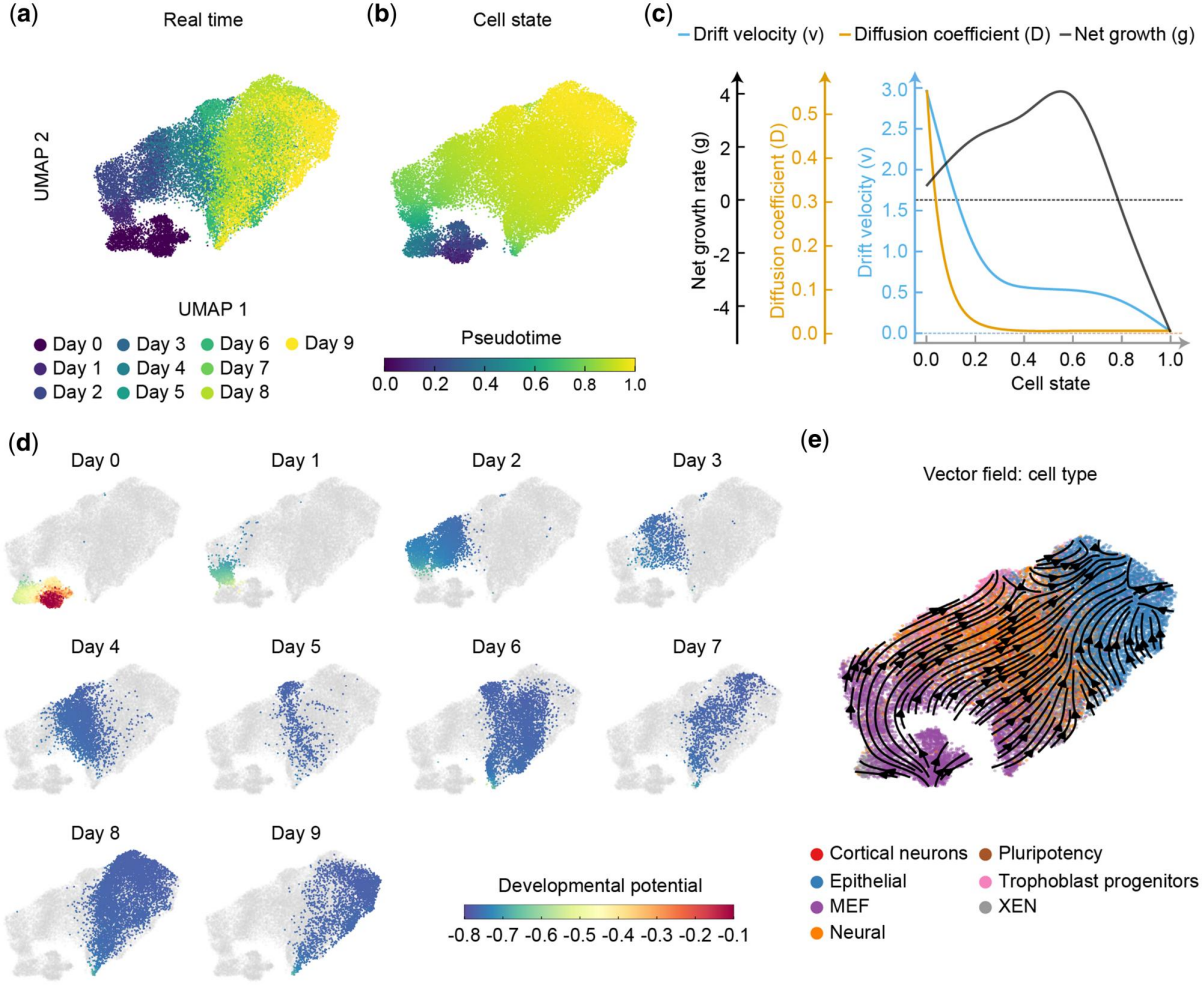
Inference of stochastic dynamics during iPSC reprogramming using scPD. (a, b) UMAP projection of the iPSC dataset, colored by (a) real sampling time (Day 0–9) and (b) diffusion pseudotime, used as the continuous cell state coordinate. (c) Inferred kinetic parameters along the cell state. Curves represent the drift velocity (*v*, blue), diffusion coefficient (*D*, yellow), and net growth rate (*g*, black). (d) Inferred developmental potential landscape projected onto the UMAP embedding across all sampled time points. (e) Reconstructed vector field derived from the inferred potential landscape. Streamlines represent the global stochastic flux, and colors indicate annotated cell types.

We next apply scPD to an iPSC reprogramming dataset sampled daily from day 0 to day 9 (Fig. 1a). Cells are ordered along a continuous cell-state coordinate using diffusion pseudotime. The inferred pseudotime shows high consistency with experimental sampling times (Fig. 1b), supporting the validity of the reconstructed trajectory. Notably, cells collected at the same time point exhibit substantial dispersion along pseudotime (Fig. S2), reflecting the intrinsic asynchrony of the reprogramming process. scPD explicitly exploits this temporal overlap by integrating discrete time-point snapshots with continuous state distributions to infer population-level dynamics.

By jointly leveraging experimental time information and continuous cell-state ordering, scPD infers state-dependent kinetic parameters that delineate distinct dynamical regimes of reprogramming (Fig. 1c). Both the drift velocity and diffusion coefficient decrease progressively along the cell-state axis and approach zero at late states, indicating a gradual loss of directed motion and stochastic variability as cells commit to terminal fates. In contrast, the net growth rate displays a non-monotonic profile: it remains positive during early and intermediate states, exhibits a transient proliferative peak, and subsequently crosses below zero at late states. This transition marks a shift from population expansion to contraction during terminal stabilization.

From these kinetic parameters, scPD further derives the developmental potential and reconstructs a high-resolution vector field. When projected onto the UMAP embedding, the inferred potential decreases monotonically along the reprogramming trajectory (Fig. 1d), consistent with an energy-minimization landscape (Wang *et al.* 2011, Weinreb *et al.* 2020). Correspondingly, the force-directed vector field accurately captures the global directionality of cell-state transitions, guiding stochastic flux from somatic MEFs through epithelial intermediates toward the pluripotent endpoint (Fig. 1e). This trajectory recapitulates the canonical mesenchymal-to-epithelial transition required for successful reprogramming, supporting the biological plausibility of the inferred dynamics (Li *et al.* 2010, Samavarchi-Tehrani *et al.* 2010).

We next assess the quantitative fidelity of the reconstructed population dynamics. At the global level, the model-predicted probability density functions closely match the empirical distributions, accurately reproducing the temporal evolution of cell-state occupancy across sampling days (Fig. S3a). For a fine-grained evaluation, model-predicted CDFs are compared with empirical CDFs at each time point (Fig. S4). Discrepancies are quantified using the area distance metric (Fig. S3b), which remains consistently low across all days, demonstrating that scPD faithfully captures both global trends and fine-scale population heterogeneity.

To assess the robustness of model fitting to smoothing choices, we evaluate the sensitivity of scPD to smoothing hyperparameters. Specifically, we vary the natural cubic spline degrees of freedom and the roughness regularization strength, and quantify distributional fitting error using the area distance across time points. The fitting error remains broadly comparable across the tested hyperparameter ranges, indicating that the distributional fit is robust to moderate changes in spline basis complexity and regularization strength (Fig. S5).

Furthermore, we address a common practical limitation of single-cell experiments: the absence of reliable time-resolved population size measurements (Fischer *et al.* 2019). To accommodate such settings, scPD provides a distribution-only mode that infers pseudotime dynamics solely from snapshot state distributions. An ablation analysis confirms the robustness of this mode: despite removing population size constraints, the inferred drift profiles and landscape topology remain highly consistent with those obtained from the full model (Fig. S6a and b). This flexibility does not compromise quantitative accuracy, as evidenced by similarly low area distance values across time points (Fig. S6c).

Lastly, we evaluate the computational efficiency of the framework. Scalability analysis via dataset downsampling (20%–100%) demonstrates that scPD efficiently processes large-scale data, scaling to over 50 000 cells in less than 10 minutes on a standard personal computer (Intel Core i7-13700H, 32 GB RAM) (Fig. S7). The numerical configuration used for the benchmark is summarized in Table S2. These results establish scPD as a robust and scalable toolkit for inferring developmental dynamics from single-cell snapshots.

## 4 Discussion and conclusion

In this work, we present scPD, a scalable Python toolkit that implements the pseudodynamics framework within the Scanpy ecosystem. By supporting both population-aware and distribution-only inference, scPD enables quantitative analysis of developmental kinetics from time-resolved single-cell snapshot data.

Despite these capabilities, scPD currently models population dynamics along a one-dimensional continuous state coordinate. This design makes the framework well suited to approximately ordered developmental processes, but it may limit the resolution of complex or multi-branching trajectories. In such cases, distinct lineage branches may be compressed onto the same state axis, making it difficult to jointly capture branch-specific transition routes, bifurcation events, or lineage-dependent kinetic profiles. For datasets with well-defined lineages, scPD can be applied separately to individual branches after branch assignment. However, extending the current framework to explicitly model branched or graph-structured state spaces remains an important direction for future development.

Overall, scPD provides an accessible framework for distribution-based model fitting, kinetic parameter inference, and landscape reconstruction in single-cell dynamical analysis. We anticipate that it will facilitate broader applications in developmental biology, regenerative medicine, and disease progression studies (Fischer *et al.* 2025, Hu *et al.* 2026).

## Supplementary Material

vbag188_Supplementary_Data

## Data Availability

The scPD package is freely available as an open-source Python package on PyPI, with source code, documentation, and example notebooks hosted on GitHub at https://github.com/yys-arch/scPD. The mouse iPSC reprogramming dataset is originally published by Schiebinger *et al.* (2019) and is available from the Gene Expression Omnibus under accession number GSE115943. The processed version used in this study, derived from the Waddington-OT tutorial resources, has been deposited on Zenodo under DOI: 10.5281/zenodo.18337517.
