# Supplementary material for: scPD: a Python package for inferring continuous population dynamics from single-cell snapshot data
Source: Bioinform Adv. 2026 Jul 1;6(1):vbag188. doi: 10.1093/bioadv/vbag188 (PMC13372687; doi:10.1093/bioadv/vbag188)
Supplement: vbag188_Supplementary_Data [file vbag188_supplementary_data.pdf]

Supplementary Information for "scPD: a Python package for inferring continuous population dynamics from single-cell snapshot data"

Supplementary note 1: model formulation

1.1 Governing dynamics

The evolution of cell density $u(s,t)$ along the normalized state coordinate $s \in [0,1]$ is governed by the one-dimensional Fokker-Planck equation shown in Eq. (1).

$$\frac{\partial u}{\partial t} = \frac{\partial}{\partial s}\left(D(s)\frac{\partial u}{\partial s}\right) - \frac{\partial}{\partial s}(v(s)u) + g(s)u. \tag{1}$$

In Eq. (1), $v(s)$ represents the drift velocity, $D(s)$ the diffusion coefficient, and $g(s)$ the net growth rate. The equation is subject to no-flux boundary conditions $J(0,t) = J(1,t) = 0$, ensuring conservation of probability mass at the boundaries.

1.2 Parameterization via natural cubic splines

To ensure smoothness and biological interpretability, we parameterize the kinetic functions $v(s)$, $D(s)$, and $g(s)$ as natural cubic splines. Each function is expressed as a linear combination of basis functions, as shown in Eq. (2).

$$f(s) = \sum_{j=1}^{K} \beta_j B_j(s). \tag{2}$$

In Eq. (2), $B_j(s)$ are the natural cubic spline basis functions with K degrees of freedom. This parameterization enforces continuity up to the second derivative and linearity beyond boundary knots.

1.3 Numerical discretization

The continuous Fokker-Planck equation is discretized using a finite volume method on a uniform grid $s_i = (i - 0.5)/N$ for $i = 1, \dots, N$. The spatial derivatives are approximated using second-order central differences for diffusion terms and upwind schemes for advection terms, yielding the semi-discrete equation in Eq. (3).

$$\frac{du_i}{dt} = \frac{1}{\Delta s}\left[J_{i-1/2} - J_{i+1/2}\right] + g_i u_i. \tag{3}$$

This yields a system of ordinary differential equations, which we solve using implicit integration schemes.

1.4 Optimization and loss function

1.4.1 Objective function

Parameter estimation is formulated as minimizing the Area Distance (L1-Wasserstein distance) between model-predicted cumulative distribution functions (CDFs) and empirical CDFs, according to Eq. (4).

$$\mathcal{L} = \sum_{k=1}^{T} \frac{A_k^2}{2\sigma_{A,k}^2}, \quad \text{with} \quad A_k = \int_0^1 |F_{\text{model}}(s,t_k) - F_{\text{emp}}(s,t_k)|ds. \tag{4}$$

In Eq. (4), $\sigma_{A,k}$ is a weighting factor derived from bootstrap resampling.

1.4.2 Regularization

To prevent overfitting, we apply the roughness penalty regularization shown in Eq. (5).

$$\mathcal{R} = \rho \sum_{f \in \{v,D,g\}} \int_0^1 \left(\frac{d^2 f}{ds^2} \right)^2 ds. \tag{5}$$

1.4.3 Fitting modes

scPD supports two fitting modes:

- **Distribution-only mode**: Fits normalized probability densities (assuming $\int u ds = 1$) by constraining the net growth term, suitable for standard scRNA-seq data lacking absolute population counts.
- **Population-aware mode**: Incorporates observed population sizes $N_{\text{obs}}(t_k)$ through an additional loss term, allowing rigorous inference of proliferation rates.

1.5 Landscape and vector field reconstruction

From the inferred drift velocity $v(s)$, we compute the developmental potential according to Eq. (6).

$$W(s) = -\int_0^s v(s')ds'. \tag{6}$$

The potential represents the quasi-potential landscape along which cells evolve. For visualization in 2D embeddings, the 1D drift is projected onto the embedding space using the local gradient of the pseudotime coordinate.

1.6 Landmark-based acceleration

For computational efficiency on large datasets ($> 2,500$ cells), scPD employs landmark-based acceleration. Cells are grouped into K landmarks using clustering (e.g., MiniBatchKMeans). The empirical distributions are then approximated using weighted kernel density estimation centered at these landmarks, as shown in Eq. (7).

$$F_{\text{landmark}}(s) \approx \sum_{k=1}^{K} w_k \mathbf{1}_{s_k \leq s}. \tag{7}$$

This reduces the computational complexity of the objective function evaluation significantly while preserving the global distributional structure.

Supplementary note 2: synthetic benchmark design

To quantitatively evaluate parameter recovery against known ground truth, we generate synthetic single-cell snapshot datasets from the same one-dimensional Fokker-Planck-type dynamics implemented in scPD. The developmental state coordinate is defined as $s \in [0, 1]$, and the ground-truth density $u(s, t)$ evolves according to Eq. (8).

$$\frac{\partial u(s,t)}{\partial t} = \frac{\partial}{\partial s}\left[D(s)\frac{\partial u(s,t)}{\partial s}\right] - \frac{\partial}{\partial s}\left[v(s)u(s,t)\right] + g(s)u(s,t). \tag{8}$$

In Eq. (8), $D(s)$ is the state-dependent diffusion coefficient, $v(s)$ is the drift velocity along the developmental coordinate, and $g(s)$ is the net growth rate. No-flux boundary conditions are imposed at $s = 0$ and $s = 1$.

For compact notation, we define the Gaussian basis function in Eq. (9).

$$G(s; \mu, \sigma) = \exp\left[-\frac{(s-\mu)^2}{2\sigma^2}\right]. \tag{9}$$

The initial density is concentrated near early developmental states, as shown in Eq. (10).

$$u(s, 0) \propto G(s; \mu_1, \sigma_1) + wG(s; \mu_2, \sigma_2). \tag{10}$$

The initial density is normalized such that $\sum_s u(s, 0)\Delta s = 1$.

We simulate the synthetic density trajectories on a cell-centered grid with 400 grid points and evaluate them at 10 discrete time points, $t = 0, 1, \ldots, 9$. We solve the equation using a finite-volume discretization with upwind advection, central diffusion, and an implicit BDF ODE solver. We compute relative population size using Eq. (11).

$$N(t) = \frac{\int_0^1 u(s,t)ds}{\int_0^1 u(s,0)ds}. \tag{11}$$

For each time point, we sample 300 pseudo single cells from the normalized density at that time point. In the main benchmark, no additional state-coordinate measurement noise or density noise is added, so that the simulated data provide a controlled test of parameter recovery under known ground-truth dynamics. We generate observed relative population sizes by applying mild log-normal noise with coefficient of variation 0.005 to the ground-truth relative population sizes.

We use two biologically motivated synthetic scenarios.

(1) Progressive differentiation toward a stable terminal state. This scenario models unidirectional differentiation from an early progenitor state toward a stable terminal state. Early states have higher drift velocity and diffusion, while terminal states have lower velocity and reduced variability. The net growth rate is mildly positive at early-to-intermediate states and decreases toward later states. The corresponding kinetic functions are defined in Eqs. (12)–(14).

$$D(s) = D_0 + D_1(1-s)^2, \tag{12}$$

$$v(s) = v_0 + v_1(1-s)^2, \tag{13}$$

$$g(s) = g_1 G(s; \mu_g, \sigma_g) - g_2 s. \tag{14}$$

(2) Transient plasticity window during fate specification. This scenario models a transient increase in cellular plasticity during fate specification. Diffusion increases around an intermediate state, drift remains forward but slows slightly near the plasticity window, and net growth contains a mild intermediate expansion with weak late depletion. The corresponding kinetic functions are defined in Eqs. (15)–(17).

$$D(s) = D_0 + D_1 G(s; \mu_D, \sigma_D), \tag{15}$$

$$v(s) = v_0 + v_1(1-s) - v_2 G(s; \mu_v, \sigma_v), \tag{16}$$

$$g(s) = g_1 G(s; \mu_{g1}, \sigma_{g1}) - g_2 G(s; \mu_{g2}, \sigma_{g2}). \tag{17}$$

The parameter values for each scenario are listed in Supplementary Table S1. These datasets provide known ground-truth functions $D(s)$, $v(s)$, and $g(s)$, allowing quantitative assessment of both distributional fitting accuracy and parameter recovery.

Supplementary tables

Table S1: **Ground-truth kinetic parameters for synthetic benchmark generation.**

Scenario	Function	Parameter values
Initial density	$u(s,0) \propto G(s;\mu_1,\sigma_1) + wG(s;\mu_2,\sigma_2)$	$\mu_1 = 0.08,\ \sigma_1 = 0.035,\ w = 0.30$<br>$\mu_2 = 0.18,\ \sigma_2 = 0.055$
Progressive differentiation	$D(s) = D_0 + D_1(1-s)^2$<br>$v(s) = v_0 + v_1(1-s)^2$<br>$g(s) = g_1G(s;\mu_g,\sigma_g) - g_2s$	$D_0 = 0.006,\ D_1 = 0.020$<br>$v_0 = 0.020,\ v_1 = 0.250$<br>$g_1 = 0.080,\ \mu_g = 0.35,\ \sigma_g = 0.18$<br>$g_2 = 0.020$
Transient plasticity window	$D(s) = D_0 + D_1G(s;\mu_D,\sigma_D)$<br><br>$v(s) = v_0 + v_1(1-s) - v_2G(s;\mu_v,\sigma_v)$<br><br>$g(s) = g_1G(s;\mu_{g1},\sigma_{g1}) - g_2G(s;\mu_{g2},\sigma_{g2})$	$D_0 = 0.008,\ D_1 = 0.035$<br>$\mu_D = 0.50,\ \sigma_D = 0.13$<br>$v_0 = 0.050,\ v_1 = 0.120,\ v_2 = 0.040$<br>$\mu_v = 0.50,\ \sigma_v = 0.15$<br>$g_1 = 0.040,\ \mu_{g1} = 0.45,\ \sigma_{g1} = 0.18$<br>$g_2 = 0.020,\ \mu_{g2} = 0.75,\ \sigma_{g2} = 0.16$

Table S2: **Numerical configuration for the scalability benchmark.**

Parameter	Benchmark setting	Description
`n_grid`	100	Number of grid points for PDE discretization
`spline_df`	4	Degrees of freedom for natural cubic spline parameterization
`n_starts`	1	Number of random optimizer initializations
`n_bootstrap`	0	Number of bootstrap samples for A-distance uncertainty estimation

Supplementary figures

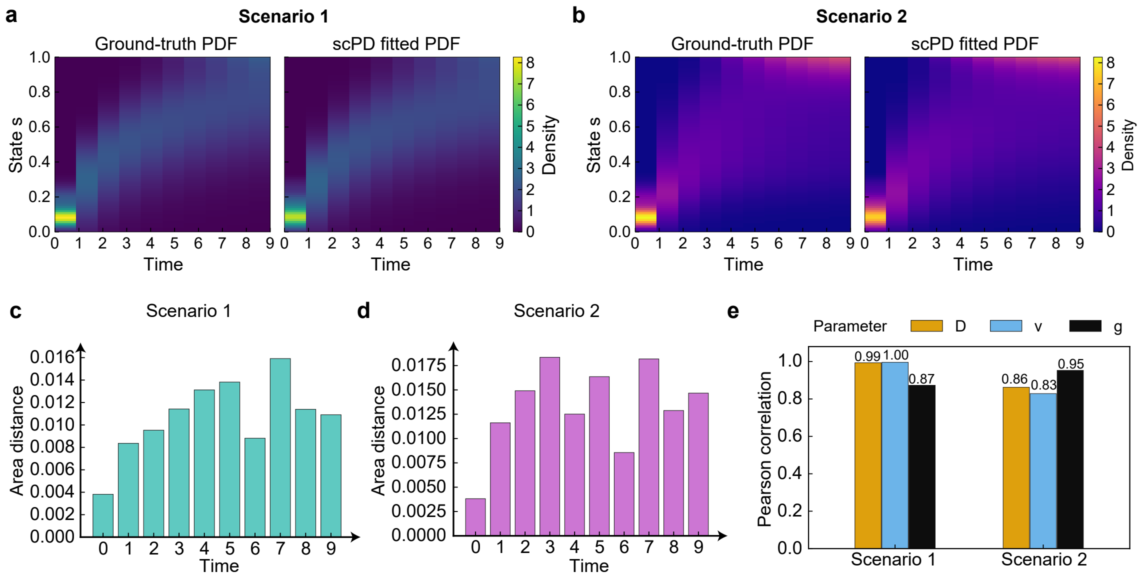

Figure S1: **Synthetic benchmark validation using known ground-truth dynamics.** (a, b) Heatmaps comparing ground-truth and scPD-fitted probability density functions (PDFs) over state coordinate s across time for Scenario 1 and Scenario 2, respectively. (c, d) Area distance between empirical and scPD-fitted cumulative distribution functions (CDFs) across time points for Scenario 1 and Scenario 2, respectively. (e) Pearson correlations between inferred and ground-truth kinetic profiles for diffusion $D(s)$, drift velocity $v(s)$, and net growth $g(s)$ in the two synthetic scenarios.

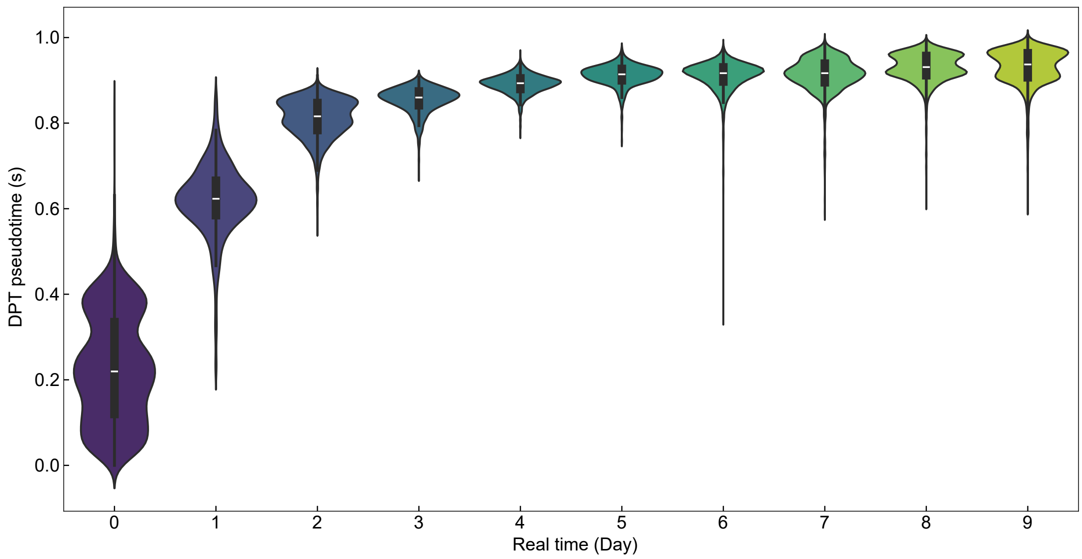

Figure S2: **Temporal distribution of cells along the inferred pseudotime trajectory.** Violin plots illustrating the density of cells along the continuous diffusion pseudotime coordinate (s) stratified by real-time sampling points (Day 0–Day 9). The white dot within each violin indicates the median value, and the thick black bar represents the interquartile range.

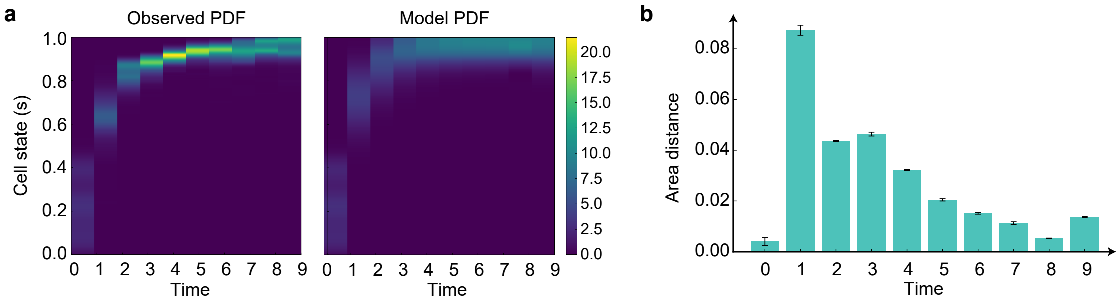

Figure S3: **Quantitative evaluation of reconstructed population dynamics during iPSC reprogramming.** (a) Comparison of the observed (left) and model-predicted (right) probability density functions (PDFs) across time and cell state. (b) Area distance values quantifying the distribution error between model and data for each time point. Error bars indicate standard deviation.

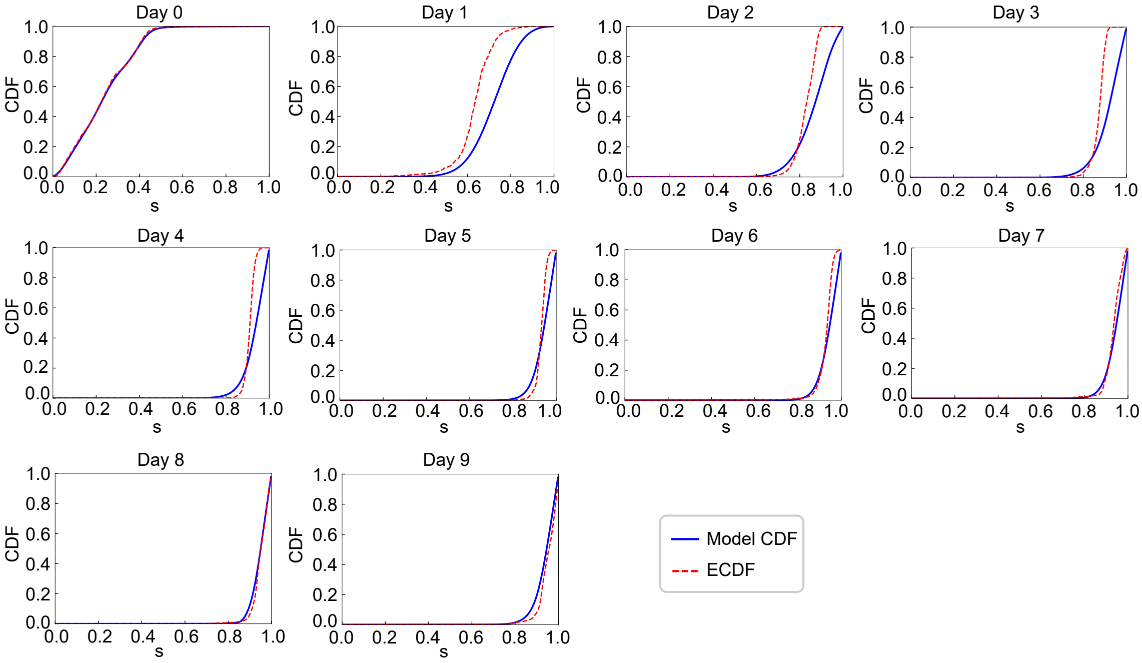

Figure S4: **Goodness-of-fit assessment via Cumulative Distribution Functions (CDFs).** Comparison between the model-predicted CDFs (blue solid lines) and the empirical CDFs (red dashed lines) derived from observed single-cell snapshots for each sampling time point (Day 0–Day 9). The close alignment between the curves indicates that the model accurately captures the cumulative probability density of the cell population at each stage.

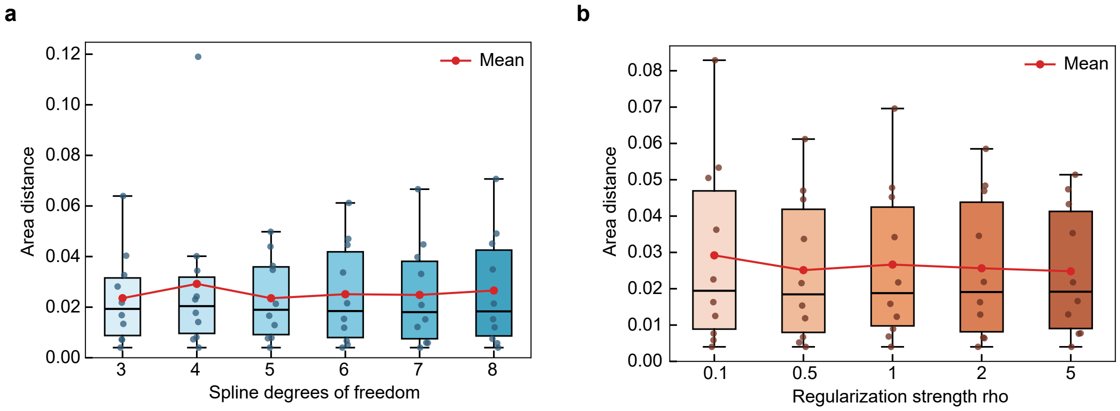

Figure S5: **Sensitivity analysis of scPD fitting error to smoothing hyperparameters.** (a) Distribution of area distance values across time points under different degrees of freedom of the natural cubic spline basis, ranging from 3 to 8. Each box summarizes area distance values across time points; dots represent individual time points, and red points/lines indicate the mean. (b) Distribution of area distance values across time points under different roughness regularization strengths, ranging from 0.1 to 5, with the spline basis degrees of freedom fixed at 6. Each box summarizes area distance values across time points; dots represent individual time points, and red points/lines indicate the mean.

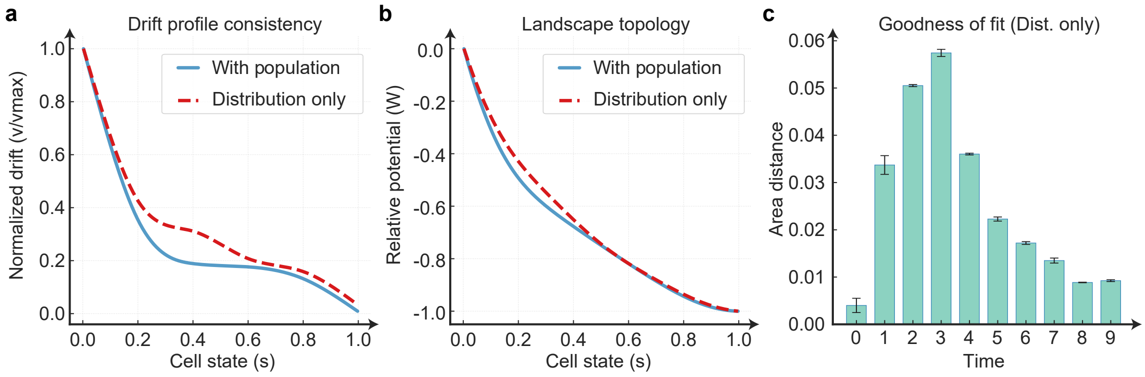

Figure S6: **Robustness analysis of the distribution-only inference mode.** (a) Comparison of the normalized drift velocity profiles (v/v_{max}) inferred using the full population-aware model (blue solid line) and the distribution-only mode (red dashed line). (b) Comparison of the reconstructed relative potential landscapes (W) between the two inference modes. (c) Bar plot quantifying the goodness-of-fit for the distribution-only mode, measured by the area distance between model-predicted and empirical distributions across all time points. Error bars represent the standard deviation.

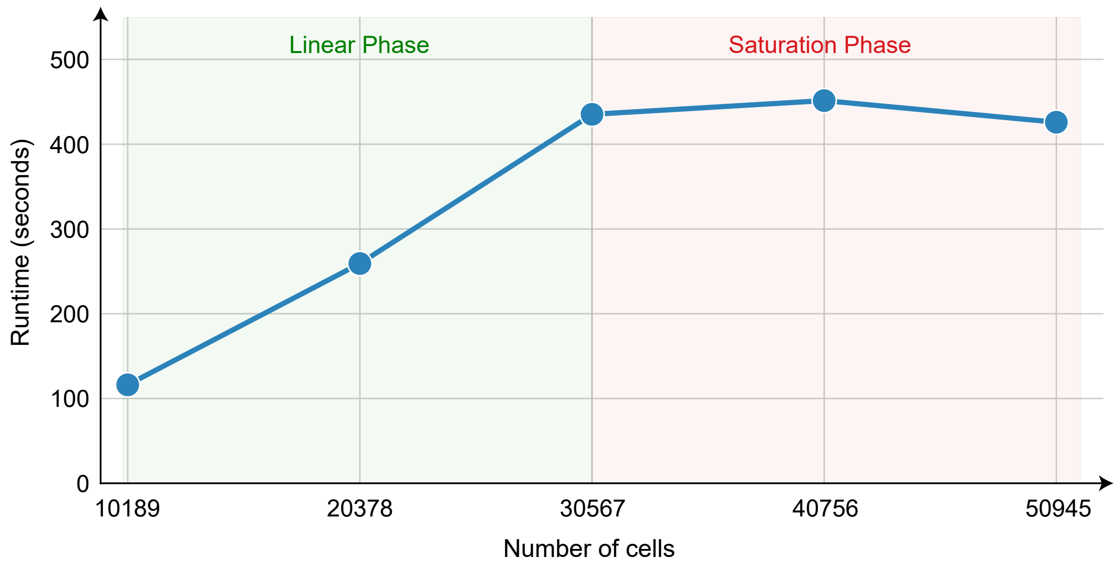

Figure S7: **Computational scalability benchmark.** Runtime performance of scPD as a function of dataset size. The iPSC dataset is downsampled to varying proportions (20%–100%, corresponding to approximately 10,000 to 51,000 cells) to evaluate computational cost. The plot displays the total inference time (in seconds) measured on a standard personal computer (Intel Core i7-13700H, 32 GB RAM). Background shading distinguishes the linear scaling phase (green) from the saturation phase (red) where landmark-based acceleration stabilizes the runtime complexity.
